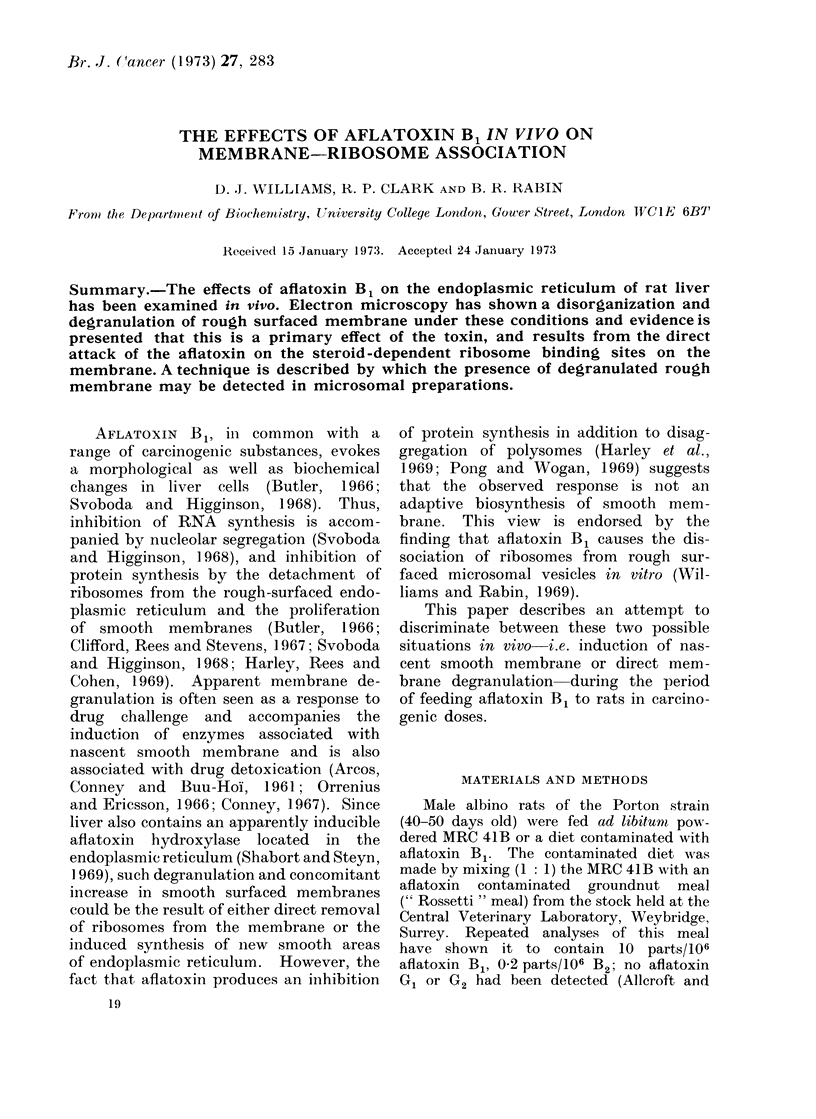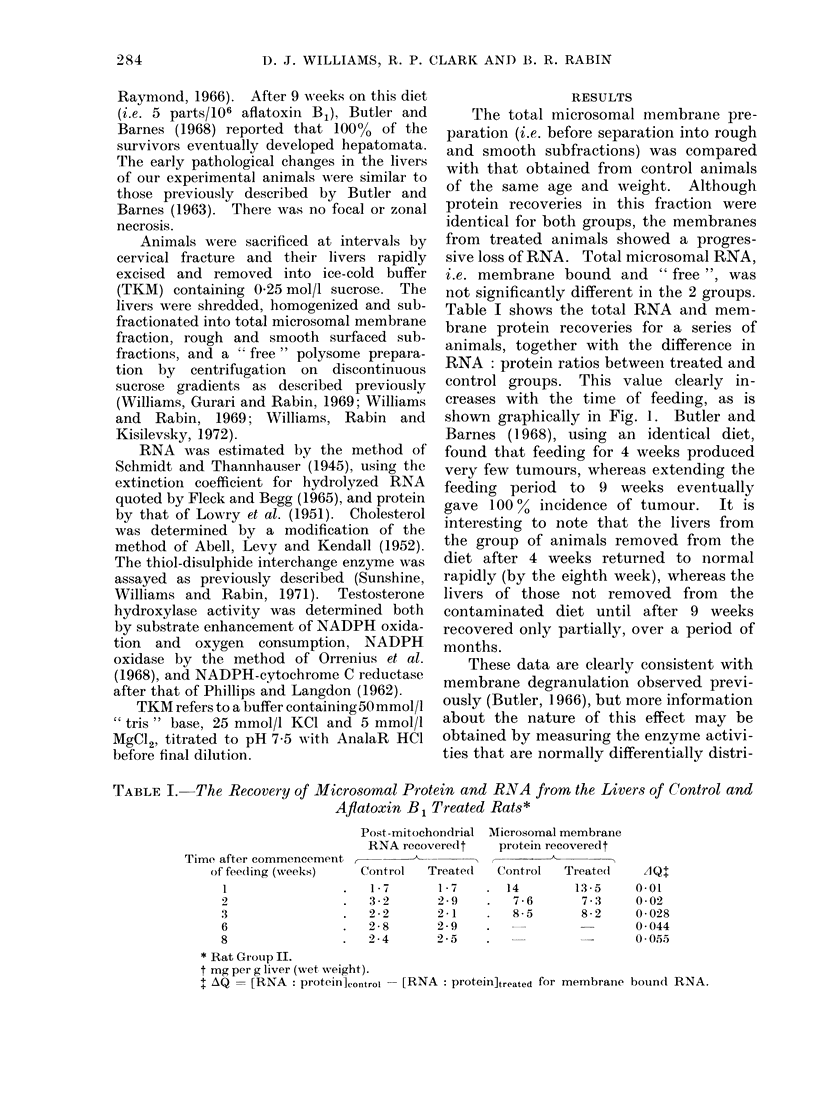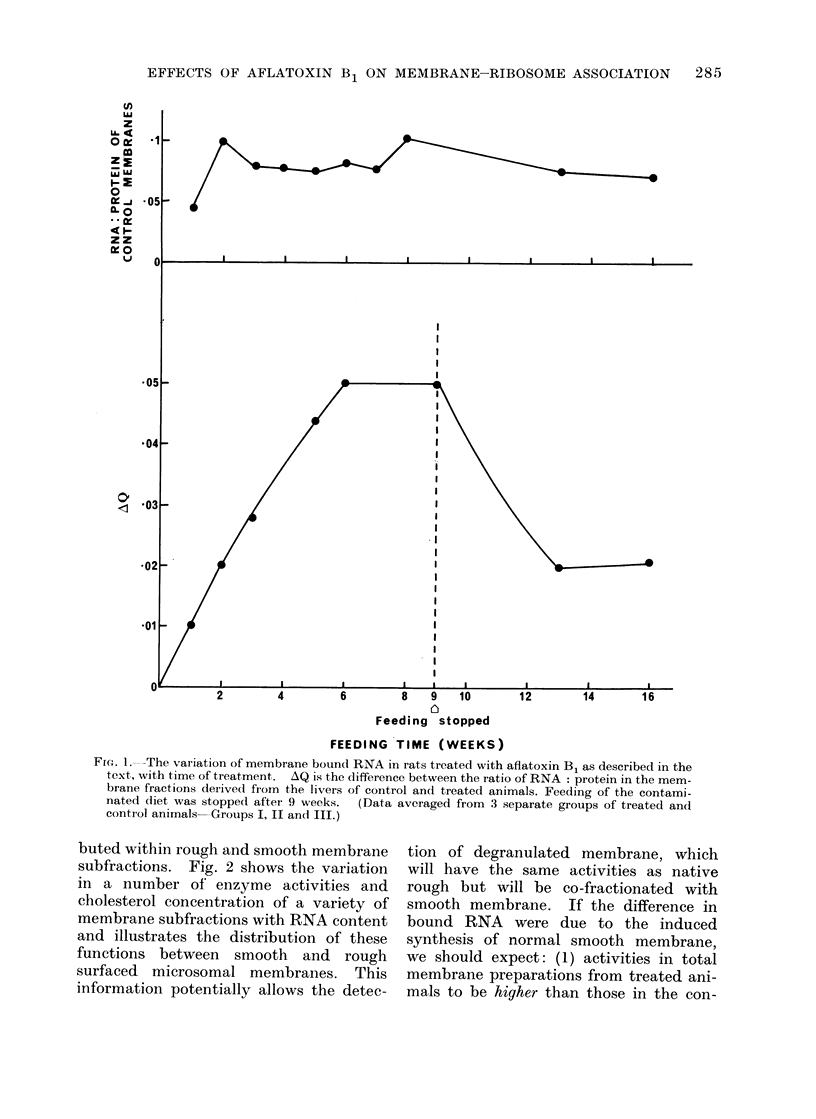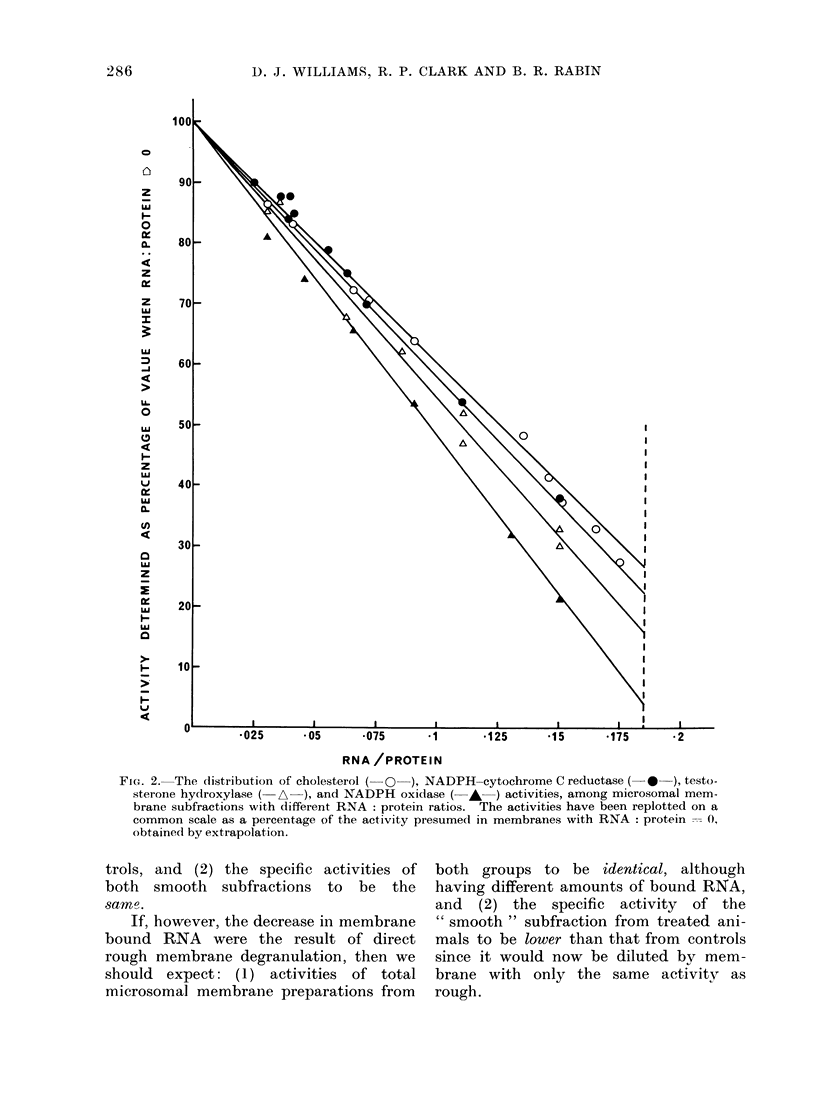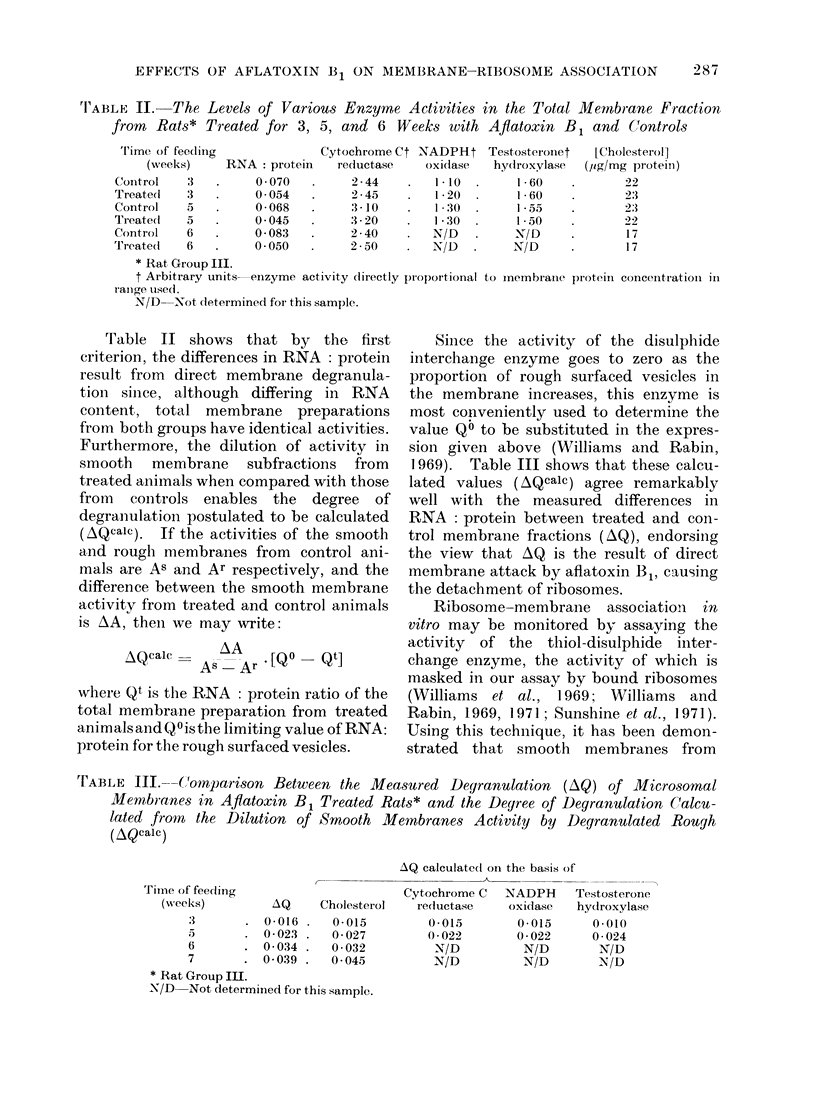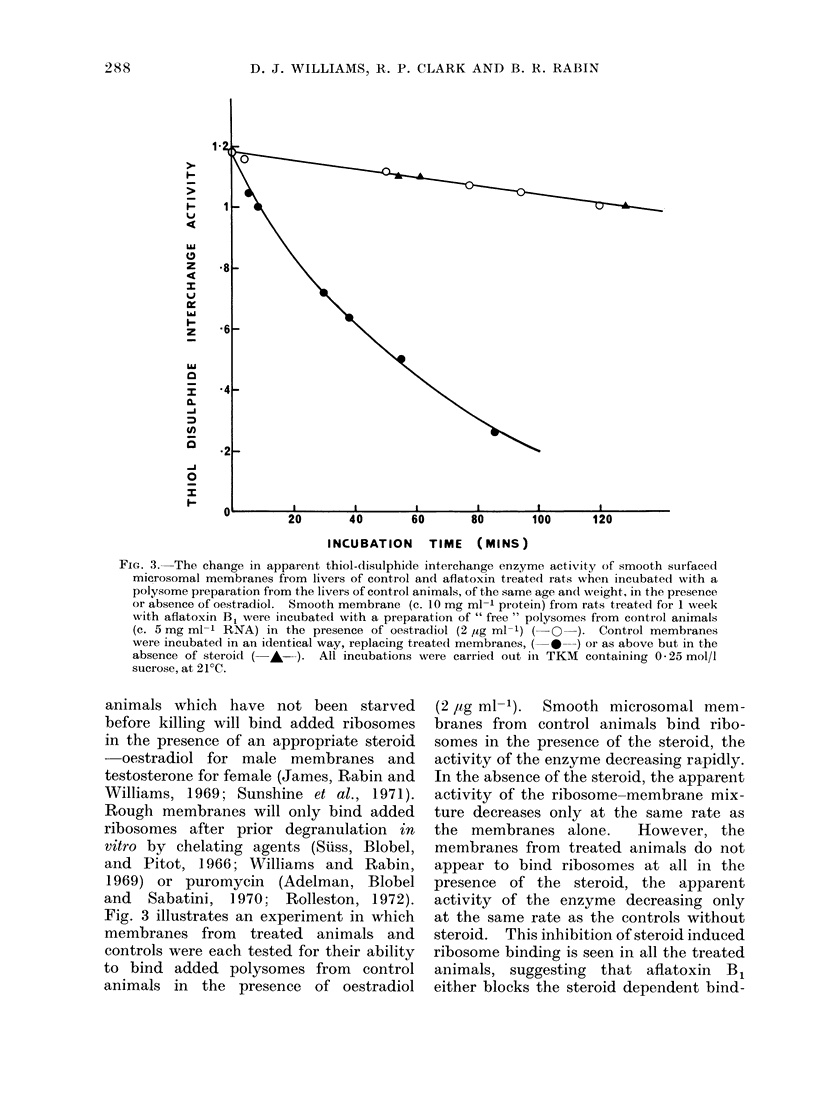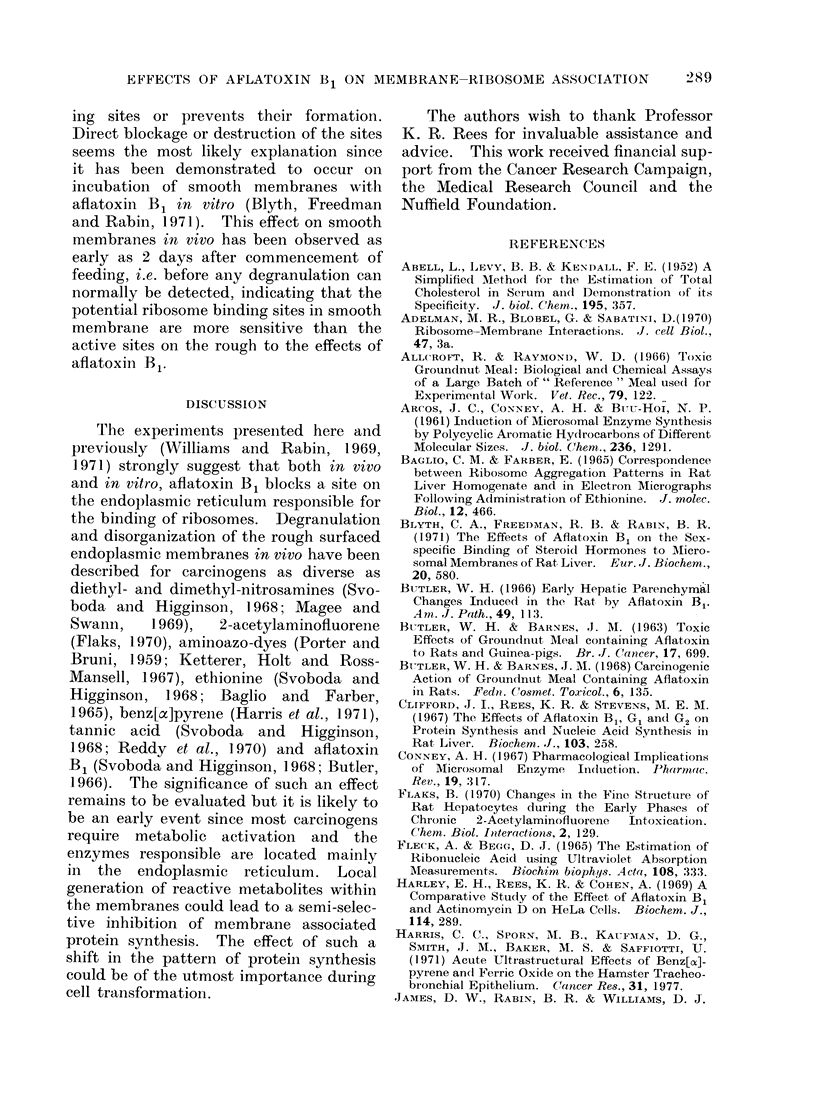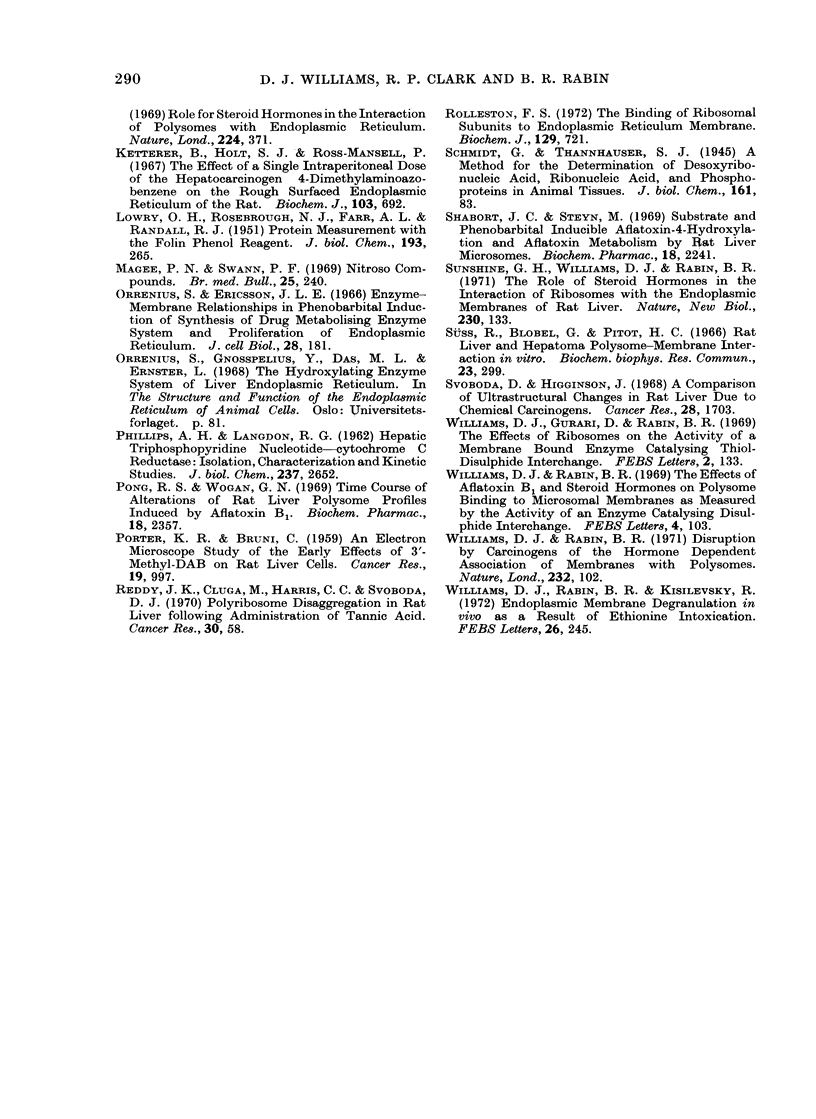# The Effects of Aflatoxin B1 in vivo on Membrane—Ribosome Association

**DOI:** 10.1038/bjc.1973.35

**Published:** 1973-04

**Authors:** D. J. Williams, R. P. Clark, B. R. Rabin

## Abstract

The effects of aflatoxin B_1_ on the endoplasmic reticulum of rat liver has been examined *in vivo.* Electron microscopy has shown a disorganization and degranulation of rough surfaced membrane under these conditions and evidence is presented that this is a primary effect of the toxin, and results from the direct attack of the aflatoxin on the steroid-dependent ribosome binding sites on the membrane. A technique is described by which the presence of degranulated rough membrane may be detected in microsomal preparations.


*Br. J. Cancer* (1973) **27**, 283

### THE EFFECTS OF AFLATOXIN $B_1$ *IN VIVO* ON MEMBRANE—RIBOSOME ASSOCIATION

D. J. WILLIAMS, R. P. CLARK AND B. R. RABIN

*From the Department of Biochemistry, University College London, Gower Street, London WC1E 6BT*

Received 15 January 1973. Accepted 24 January 1973

**Summary.—The effects of aflatoxin $B_1$ on the endoplasmic reticulum of rat liver has been examined *in vivo*. Electron microscopy has shown a disorganization and degranulation of rough surfaced membrane under these conditions and evidence is presented that this is a primary effect of the toxin, and results from the direct attack of the aflatoxin on the steroid-dependent ribosome binding sites on the membrane. A technique is described by which the presence of degranulated rough membrane may be detected in microsomal preparations.**

AFLATOXIN $B_1$, in common with a range of carcinogenic substances, evokes a morphological as well as biochemical changes in liver cells (Butler, 1966; Svoboda and Higginson, 1968). Thus, inhibition of RNA synthesis is accompanied by nucleolar segregation (Svoboda and Higginson, 1968), and inhibition of protein synthesis by the detachment of ribosomes from the rough-surfaced endoplasmic reticulum and the proliferation of smooth membranes (Butler, 1966; Clifford, Rees and Stevens, 1967; Svoboda and Higginson, 1968; Harley, Rees and Cohen, 1969). Apparent membrane degranulation is often seen as a response to drug challenge and accompanies the induction of enzymes associated with nascent smooth membrane and is also associated with drug detoxication (Arcos, Conney and Buu-Hoï, 1961; Orrenius and Ericsson, 1966; Conney, 1967). Since liver also contains an apparently inducible aflatoxin hydroxylase located in the endoplasmic reticulum (Shabort and Steyn, 1969), such degranulation and concomitant increase in smooth surfaced membranes could be the result of either direct removal of ribosomes from the membrane or the induced synthesis of new smooth areas of endoplasmic reticulum. However, the fact that aflatoxin produces an inhibition of protein synthesis in addition to disaggregation of polysomes (Harley *et al.*, 1969; Pong and Wogan, 1969) suggests that the observed response is not an adaptive biosynthesis of smooth membrane. This view is endorsed by the finding that aflatoxin $B_1$ causes the dissociation of ribosomes from rough surfaced microsomal vesicles *in vitro* (Williams and Rabin, 1969).

This paper describes an attempt to discriminate between these two possible situations *in vivo*—*i.e.* induction of nascent smooth membrane or direct membrane degranulation—during the period of feeding aflatoxin $B_1$ to rats in carcinogenic doses.

### MATERIALS AND METHODS

Male albino rats of the Porton strain (40–50 days old) were fed *ad libitum* powdered MRC 41B or a diet contaminated with aflatoxin $B_1$. The contaminated diet was made by mixing (1 : 1) the MRC 41B with an aflatoxin contaminated groundnut meal (" Rossetti " meal) from the stock held at the Central Veterinary Laboratory, Weybridge, Surrey. Repeated analyses of this meal have shown it to contain 10 parts/$10^6$ aflatoxin $B_1$, 0·2 parts/$10^6$ $B_2$; no aflatoxin $G_1$ or $G_2$ had been detected (Allcroft and

Raymond, 1966). After 9 weeks on this diet (*i.e.* 5 parts/$10^6$ aflatoxin $B_1$), Butler and Barnes (1968) reported that 100% of the survivors eventually developed hepatomata. The early pathological changes in the livers of our experimental animals were similar to those previously described by Butler and Barnes (1963). There was no focal or zonal necrosis.

Animals were sacrificed at intervals by cervical fracture and their livers rapidly excised and removed into ice-cold buffer (TKM) containing 0·25 mol/l sucrose. The livers were shredded, homogenized and subfractionated into total microsomal membrane fraction, rough and smooth surfaced subfractions, and a " free " polysome preparation by centrifugation on discontinuous sucrose gradients as described previously (Williams, Gurari and Rabin, 1969; Williams and Rabin, 1969; Williams, Rabin and Kisilevsky, 1972).

RNA was estimated by the method of Schmidt and Thannhauser (1945), using the extinction coefficient for hydrolyzed RNA quoted by Fleck and Begg (1965), and protein by that of Lowry *et al.* (1951). Cholesterol was determined by a modification of the method of Abell, Levy and Kendall (1952). The thiol-disulphide interchange enzyme was assayed as previously described (Sunshine, Williams and Rabin, 1971). Testosterone hydroxylase activity was determined both by substrate enhancement of NADPH oxidation and oxygen consumption, NADPH oxidase by the method of Orrenius *et al.* (1968), and NADPH-cytochrome C reductase after that of Phillips and Langdon (1962).

TKM refers to a buffer containing 50 mmol/l " tris " base, 25 mmol/l KCl and 5 mmol/l $MgCl_2$, titrated to pH 7·5 with AnalaR HCl before final dilution.

### RESULTS

The total microsomal membrane preparation (*i.e.* before separation into rough and smooth subfractions) was compared with that obtained from control animals of the same age and weight. Although protein recoveries in this fraction were identical for both groups, the membranes from treated animals showed a progressive loss of RNA. Total microsomal RNA, *i.e.* membrane bound and " free ", was not significantly different in the 2 groups. Table I shows the total RNA and membrane protein recoveries for a series of animals, together with the difference in RNA : protein ratios between treated and control groups. This value clearly increases with the time of feeding, as is shown graphically in Fig. 1. Butler and Barnes (1968), using an identical diet, found that feeding for 4 weeks produced very few tumours, whereas extending the feeding period to 9 weeks eventually gave 100% incidence of tumour. It is interesting to note that the livers from the group of animals removed from the diet after 4 weeks returned to normal rapidly (by the eighth week), whereas the livers of those not removed from the contaminated diet until after 9 weeks recovered only partially, over a period of months.

These data are clearly consistent with membrane degranulation observed previously (Butler, 1966), but more information about the nature of this effect may be obtained by measuring the enzyme activities that are normally differentially distri-

TABLE I.—*The Recovery of Microsomal Protein and RNA from the Livers of Control and Aflatoxin $B_1$ Treated Rats**

| Time after commencement of feeding (weeks) | Post-mitochondrial RNA recovered† | | Microsomal membrane protein recovered† | | $\Delta Q$‡ |
|---|---|---|---|---|---|
| | Control | Treated | Control | Treated | |
| 1 | 1·7 | 1·7 | 14 | 13·5 | 0·01 |
| 2 | 3·2 | 2·9 | 7·6 | 7·3 | 0·02 |
| 3 | 2·2 | 2·1 | 8·5 | 8·2 | 0·028 |
| 6 | 2·8 | 2·9 | — | — | 0·044 |
| 8 | 2·4 | 2·5 | — | — | 0·055 |

* Rat Group II.

† mg per g liver (wet weight).

‡ $\Delta Q = [\text{RNA : protein}]_{control} - [\text{RNA : protein}]_{treated}$ for membrane bound RNA.

FIG. 1.—The variation of membrane bound RNA in rats treated with aflatoxin $B_1$ as described in the text, with time of treatment. ΔQ is the difference between the ratio of RNA : protein in the membrane fractions derived from the livers of control and treated animals. Feeding of the contaminated diet was stopped after 9 weeks. (Data averaged from 3 separate groups of treated and control animals—Groups I, II and III.)

buted within rough and smooth membrane subfractions. Fig. 2 shows the variation in a number of enzyme activities and cholesterol concentration of a variety of membrane subfractions with RNA content and illustrates the distribution of these functions between smooth and rough surfaced microsomal membranes. This information potentially allows the detection of degranulated membrane, which will have the same activities as native rough but will be co-fractionated with smooth membrane. If the difference in bound RNA were due to the induced synthesis of normal smooth membrane, we should expect: (1) activities in total membrane preparations from treated animals to be *higher* than those in the con-

Fig. 2.—The distribution of cholesterol (—○—), NADPH–cytochrome C reductase (—●—), testosterone hydroxylase (—△—), and NADPH oxidase (—▲—) activities, among microsomal membrane subfractions with different RNA : protein ratios. The activities have been replotted on a common scale as a percentage of the activity presumed in membranes with RNA : protein = 0, obtained by extrapolation.

trols, and (2) the specific activities of both smooth subfractions to be the *same*.

If, however, the decrease in membrane bound RNA were the result of direct rough membrane degranulation, then we should expect: (1) activities of total microsomal membrane preparations from both groups to be *identical*, although having different amounts of bound RNA, and (2) the specific activity of the " smooth " subfraction from treated animals to be *lower* than that from controls since it would now be diluted by membrane with only the same activity as rough.

TABLE II.—*The Levels of Various Enzyme Activities in the Total Membrane Fraction from Rats* Treated for 3, 5, and 6 Weeks with Aflatoxin $B_1$ and Controls*

| Time of feeding (weeks) | | RNA : protein | Cytochrome C† reductase | NADPH† oxidase | Testosterone† hydroxylase | [Cholesterol] (μg/mg protein) |
|---|---|---|---|---|---|---|
| Control | 3 | 0·070 | 2·44 | 1·10 | 1·60 | 22 |
| Treated | 3 | 0·054 | 2·45 | 1·20 | 1·60 | 23 |
| Control | 5 | 0·068 | 3·10 | 1·30 | 1·55 | 23 |
| Treated | 5 | 0·045 | 3·20 | 1·30 | 1·50 | 22 |
| Control | 6 | 0·083 | 2·40 | N/D | N/D | 17 |
| Treated | 6 | 0·050 | 2·50 | N/D | N/D | 17 |

* Rat Group III.

† Arbitrary units—enzyme activity directly proportional to membrane protein concentration in range used.

N/D—Not determined for this sample.

Table II shows that by the first criterion, the differences in RNA : protein result from direct membrane degranulation since, although differing in RNA content, total membrane preparations from both groups have identical activities. Furthermore, the dilution of activity in smooth membrane subfractions from treated animals when compared with those from controls enables the degree of degranulation postulated to be calculated ($\Delta Q^{calc}$). If the activities of the smooth and rough membranes from control animals are $A^s$ and $A^r$ respectively, and the difference between the smooth membrane activity from treated and control animals is $\Delta A$, then we may write:

$$\Delta Q^{calc} = \frac{\Delta A}{A^s - A^r} \cdot [Q^0 - Q^t]$$

where $Q^t$ is the RNA : protein ratio of the total membrane preparation from treated animals and $Q^0$ is the limiting value of RNA: protein for the rough surfaced vesicles.

Since the activity of the disulphide interchange enzyme goes to zero as the proportion of rough surfaced vesicles in the membrane increases, this enzyme is most conveniently used to determine the value $Q^0$ to be substituted in the expression given above (Williams and Rabin, 1969). Table III shows that these calculated values ($\Delta Q^{calc}$) agree remarkably well with the measured differences in RNA : protein between treated and control membrane fractions ($\Delta Q$), endorsing the view that $\Delta Q$ is the result of direct membrane attack by aflatoxin $B_1$, causing the detachment of ribosomes.

Ribosome–membrane association *in vitro* may be monitored by assaying the activity of the thiol-disulphide interchange enzyme, the activity of which is masked in our assay by bound ribosomes (Williams *et al.*, 1969; Williams and Rabin, 1969, 1971; Sunshine *et al.*, 1971). Using this technique, it has been demonstrated that smooth membranes from

TABLE III.—*Comparison Between the Measured Degranulation ($\Delta Q$) of Microsomal Membranes in Aflatoxin $B_1$ Treated Rats* and the Degree of Degranulation Calculated from the Dilution of Smooth Membranes Activity by Degranulated Rough ($\Delta Q^{calc}$)*

| Time of feeding (weeks) | ΔQ | ΔQ calculated on the basis of: Cholesterol | Cytochrome C reductase | NADPH oxidase | Testosterone hydroxylase |
|---|---|---|---|---|---|
| 3 | 0·016 | 0·015 | 0·015 | 0·015 | 0·010 |
| 5 | 0·023 | 0·027 | 0·022 | 0·022 | 0·024 |
| 6 | 0·034 | 0·032 | N/D | N/D | N/D |
| 7 | 0·039 | 0·045 | N/D | N/D | N/D |

* Rat Group III.
N/D—Not determined for this sample.

FIG. 3.—The change in apparent thiol-disulphide interchange enzyme activity of smooth surfaced microsomal membranes from livers of control and aflatoxin treated rats when incubated with a polysome preparation from the livers of control animals, of the same age and weight, in the presence or absence of oestradiol. Smooth membrane (c. 10 mg $ml^{-1}$ protein) from rats treated for 1 week with aflatoxin $B_1$ were incubated with a preparation of " free " polysomes from control animals (c. 5 mg $ml^{-1}$ RNA) in the presence of oestradiol (2 $\mu g$ $ml^{-1}$) (—○—). Control membranes were incubated in an identical way, replacing treated membranes, (—●—) or as above but in the absence of steroid (—▲—). All incubations were carried out in TKM containing 0·25 mol/l sucrose, at 21°C.

animals which have not been starved before killing will bind added ribosomes in the presence of an appropriate steroid —oestradiol for male membranes and testosterone for female (James, Rabin and Williams, 1969; Sunshine *et al.*, 1971). Rough membranes will only bind added ribosomes after prior degranulation *in vitro* by chelating agents (Süss, Blobel, and Pitot, 1966; Williams and Rabin, 1969) or puromycin (Adelman, Blobel and Sabatini, 1970; Rolleston, 1972). Fig. 3 illustrates an experiment in which membranes from treated animals and controls were each tested for their ability to bind added polysomes from control animals in the presence of oestradiol (2 $\mu g$ $ml^{-1}$). Smooth microsomal membranes from control animals bind ribosomes in the presence of the steroid, the activity of the enzyme decreasing rapidly. In the absence of the steroid, the apparent activity of the ribosome–membrane mixture decreases only at the same rate as the membranes alone. However, the membranes from treated animals do not appear to bind ribosomes at all in the presence of the steroid, the apparent activity of the enzyme decreasing only at the same rate as the controls without steroid. This inhibition of steroid induced ribosome binding is seen in all the treated animals, suggesting that aflatoxin $B_1$ either blocks the steroid dependent binding sites or prevents their formation. Direct blockage or destruction of the sites seems the most likely explanation since it has been demonstrated to occur on incubation of smooth membranes with aflatoxin $B_1$ *in vitro* (Blyth, Freedman and Rabin, 1971). This effect on smooth membranes *in vivo* has been observed as early as 2 days after commencement of feeding, *i.e.* before any degranulation can normally be detected, indicating that the potential ribosome binding sites in smooth membrane are more sensitive than the active sites on the rough to the effects of aflatoxin $B_1$.

### DISCUSSION

The experiments presented here and previously (Williams and Rabin, 1969, 1971) strongly suggest that both *in vivo* and *in vitro*, aflatoxin $B_1$ blocks a site on the endoplasmic reticulum responsible for the binding of ribosomes. Degranulation and disorganization of the rough surfaced endoplasmic membranes *in vivo* have been described for carcinogens as diverse as diethyl- and dimethyl-nitrosamines (Svoboda and Higginson, 1968; Magee and Swann, 1969), 2-acetylaminofluorene (Flaks, 1970), aminoazo-dyes (Porter and Bruni, 1959; Ketterer, Holt and Ross-Mansell, 1967), ethionine (Svoboda and Higginson, 1968; Baglio and Farber, 1965), benz[α]pyrene (Harris *et al.*, 1971), tannic acid (Svoboda and Higginson, 1968; Reddy *et al.*, 1970) and aflatoxin $B_1$ (Svoboda and Higginson, 1968; Butler, 1966). The significance of such an effect remains to be evaluated but it is likely to be an early event since most carcinogens require metabolic activation and the enzymes responsible are located mainly in the endoplasmic reticulum. Local generation of reactive metabolites within the membranes could lead to a semi-selective inhibition of membrane associated protein synthesis. The effect of such a shift in the pattern of protein synthesis could be of the utmost importance during cell transformation.

The authors wish to thank Professor K. R. Rees for invaluable assistance and advice. This work received financial support from the Cancer Research Campaign, the Medical Research Council and the Nuffield Foundation.

### REFERENCES

ABELL, L., LEVY, B. B. & KENDALL, F. E. (1952) A Simplified Method for the Estimation of Total Cholesterol in Serum and Demonstration of its Specificity. *J. biol. Chem.*, **195**, 357.

ADELMAN, M. R., BLOBEL, G. & SABATINI, D.(1970) Ribosome-Membrane Interactions. *J. cell Biol.*, **47**, 3a.

ALLCROFT, R. & RAYMOND, W. D. (1966) Toxic Groundnut Meal: Biological and Chemical Assays of a Large Batch of " Reference " Meal used for Experimental Work. *Vet. Rec.*, **79**, 122.

ARCOS, J. C., CONNEY, A. H. & BUU-HOÏ, N. P. (1961) Induction of Microsomal Enzyme Synthesis by Polycyclic Aromatic Hydrocarbons of Different Molecular Sizes. *J. biol. Chem.*, **236**, 1291.

BAGLIO, C. M. & FARBER, E. (1965) Correspondence between Ribosome Aggregation Patterns in Rat Liver Homogenate and in Electron Micrographs Following Administration of Ethionine. *J. molec. Biol.*, **12**, 466.

BLYTH, C. A., FREEDMAN, R. B. & RABIN, B. R. (1971) The Effects of Aflatoxin $B_1$ on the Sex-specific Binding of Steroid Hormones to Microsomal Membranes of Rat Liver. *Eur. J. Biochem.*, **20**, 580.

BUTLER, W. H. (1966) Early Hepatic Parenchymal Changes Induced in the Rat by Aflatoxin $B_1$. *Am. J. Path.*, **49**, 113.

BUTLER, W. H. & BARNES, J. M. (1963) Toxic Effects of Groundnut Meal containing Aflatoxin to Rats and Guinea-pigs. *Br. J. Cancer*, **17**, 699.

BUTLER, W. H. & BARNES, J. M. (1968) Carcinogenic Action of Groundnut Meal Containing Aflatoxin in Rats. *Fedn. Cosmet. Toxicol.*, **6**, 135.

CLIFFORD, J. I., REES, K. R. & STEVENS, M. E. M. (1967) The Effects of Aflatoxin $B_1$, $G_1$ and $G_2$ on Protein Synthesis and Nucleic Acid Synthesis in Rat Liver. *Biochem. J.*, **103**, 258.

CONNEY, A. H. (1967) Pharmacological Implications of Microsomal Enzyme Induction. *Pharmac. Rev.*, **19**, 317.

FLAKS, B. (1970) Changes in the Fine Structure of Rat Hepatocytes during the Early Phases of Chronic 2-Acetylaminofluorene Intoxication. *Chem. Biol. Interactions*, **2**, 129.

FLECK, A. & BEGG, D. J. (1965) The Estimation of Ribonucleic Acid using Ultraviolet Absorption Measurements. *Biochim biophys. Acta*, **108**, 333.

HARLEY, E. H., REES, K. R. & COHEN, A. (1969) A Comparative Study of the Effect of Aflatoxin $B_1$ and Actinomycin D on HeLa Cells. *Biochem. J.*, **114**, 289.

HARRIS, C. C., SPORN, M. B., KAUFMAN, D. G., SMITH, J. M., BAKER, M. S. & SAFFIOTTI, U. (1971) Acute Ultrastructural Effects of Benz[α]-pyrene and Ferric Oxide on the Hamster Tracheobronchial Epithelium. *Cancer Res.*, **31**, 1977.

JAMES, D. W., RABIN, B. R. & WILLIAMS, D. J.

(1969) Role for Steroid Hormones in the Interaction of Polysomes with Endoplasmic Reticulum. *Nature, Lond.*, **224**, 371.

KETTERER, B., HOLT, S. J. & ROSS-MANSELL, P. (1967) The Effect of a Single Intraperitoneal Dose of the Hepatocarcinogen 4-Dimethylaminoazobenzene on the Rough Surfaced Endoplasmic Reticulum of the Rat. *Biochem. J.*, **103**, 692.

LOWRY, O. H., ROSEBROUGH, N. J., FARR, A. L. & RANDALL, R. J. (1951) Protein Measurement with the Folin Phenol Reagent. *J. biol. Chem.*, **193**, 265.

MAGEE, P. N. & SWANN, P. F. (1969) Nitroso Compounds. *Br. med. Bull.*, **25**, 240.

ORRENIUS, S. & ERICSSON, J. L. E. (1966) Enzyme–Membrane Relationships in Phenobarbital Induction of Synthesis of Drug Metabolising Enzyme System and Proliferation of Endoplasmic Reticulum. *J. cell Biol.*, **28**, 181.

ORRENIUS, S., GNOSSPELIUS, Y., DAS, M. L. & ERNSTER, L. (1968) The Hydroxylating Enzyme System of Liver Endoplasmic Reticulum. In *The Structure and Function of the Endoplasmic Reticulum of Animal Cells.* Oslo: Universitetsforlaget. p. 81.

PHILLIPS, A. H. & LANGDON, R. G. (1962) Hepatic Triphosphopyridine Nucleotide—cytochrome C Reductase: Isolation, Characterization and Kinetic Studies. *J. biol. Chem.*, **237**, 2652.

PONG, R. S. & WOGAN, G. N. (1969) Time Course of Alterations of Rat Liver Polysome Profiles Induced by Aflatoxin $B_1$. *Biochem. Pharmac.*, **18**, 2357.

PORTER, K. R. & BRUNI, C. (1959) An Electron Microscope Study of the Early Effects of 3′-Methyl-DAB on Rat Liver Cells. *Cancer Res.*, **19**, 997.

REDDY, J. K., CLUGA, M., HARRIS, C. C. & SVOBODA, D. J. (1970) Polyribosome Disaggregation in Rat Liver following Administration of Tannic Acid. *Cancer Res.*, **30**, 58.

ROLLESTON, F. S. (1972) The Binding of Ribosomal Subunits to Endoplasmic Reticulum Membrane. *Biochem. J.*, **129**, 721.

SCHMIDT, G. & THANNHAUSER, S. J. (1945) A Method for the Determination of Desoxyribonucleic Acid, Ribonucleic Acid, and Phosphoproteins in Animal Tissues. *J. biol. Chem.*, **161**, 83.

SHABORT, J. C. & STEYN, M. (1969) Substrate and Phenobarbital Inducible Aflatoxin-4-Hydroxylation and Aflatoxin Metabolism by Rat Liver Microsomes. *Biochem. Pharmac.*, **18**, 2241.

SUNSHINE, G. H., WILLIAMS, D. J. & RABIN, B. R. (1971) The Role of Steroid Hormones in the Interaction of Ribosomes with the Endoplasmic Membranes of Rat Liver. *Nature, New Biol.*, **230**, 133.

SÜSS, R., BLOBEL, G. & PITOT, H. C. (1966) Rat Liver and Hepatoma Polysome–Membrane Interaction *in vitro*. *Biochem. biophys. Res. Commun.*, **23**, 299.

SVOBODA, D. & HIGGINSON, J. (1968) A Comparison of Ultrastructural Changes in Rat Liver Due to Chemical Carcinogens. *Cancer Res.*, **28**, 1703.

WILLIAMS, D. J., GURARI, D. & RABIN, B. R. (1969) The Effects of Ribosomes on the Activity of a Membrane Bound Enzyme Catalysing Thiol-Disulphide Interchange. *FEBS Letters*, **2**, 133.

WILLIAMS, D. J. & RABIN, B. R. (1969) The Effects of Aflatoxin $B_1$ and Steroid Hormones on Polysome Binding to Microsomal Membranes as Measured by the Activity of an Enzyme Catalysing Disulphide Interchange. *FEBS Letters*, **4**, 103.

WILLIAMS, D. J. & RABIN, B. R. (1971) Disruption by Carcinogens of the Hormone Dependent Association of Membranes with Polysomes. *Nature, Lond.*, **232**, 102.

WILLIAMS, D. J., RABIN, B. R. & KISILEVSKY, R. (1972) Endoplasmic Membrane Degranulation *in vivo* as a Result of Ethionine Intoxication. *FEBS Letters*, **26**, 245.